# Resveratrol and Immune Cells: A Link to Improve Human Health

**DOI:** 10.3390/molecules27020424

**Published:** 2022-01-10

**Authors:** Alessio Alesci, Noemi Nicosia, Angelo Fumia, Federica Giorgianni, Antonello Santini, Nicola Cicero

**Affiliations:** 1 Department of Chemical, Biological, Pharmaceutical and Environmental Sciences, University of Messina, Viale Stagno d’Alcontres, 31, 98166 Messina, Italy; alessio.alesci@gmail.com (A.A.); noemi.nicosia92@gmail.com (N.N.); 2 Foundation “Prof. Antonio Imbesi”, University of Messina, Piazza Pugliatti 1, 98122 Messina, Italy; 3 Department of Pharmacological Screening, Medical College, Jagiellonian University, Medyczna 9, PL 30-688 Cracow, Poland; 4 Department of Clinical and Experimental Medicine, University of Messina, Padiglione C, A. O. U. Policlinico “G. Martino”, Viale Gazzi, 98147 Messina, Italy; angelo.fumia@gmail.com; 5 Department of Biomedical and Dental Science and Morphofunctional Imaging, University of Messina, Via Consolare Valeria, 98125 Messina, Italy; giorgiannifederica1@gmail.com (F.G.); ncicero@unime.it (N.C.); 6 Department of Pharmacy, University of Napoli Federico II, Via D. Montesano 49, 80131 Napoli, Italy; 7 Science4life Spin-off Company, University of Messina, 98168 Messina, Italy; 8 Consorzio di Ricerca sul Rischio Biologico in Agricoltura (Co.Ri.Bi.A), 90129 Palermo, Italy

**Keywords:** resveratrol, immune cells, nutraceuticals, SIRT1, inflammation

## Abstract

The use of polyphenols as adjuvants in lowering risk factors for various debilitating diseases has been investigated in recent years due to their possible antioxidant action. Polyphenols represent a fascinating and relatively new subject of research in nutraceuticals and nutrition, with interest rapidly expanding since they can help maintain health by controlling metabolism, weight, chronic diseases, and cell proliferation. Resveratrol is a phenolic compound found mostly in the pulp, peels, seeds, and stems of red grapes. It has a wide variety of biological actions that can be used to prevent the beginning of various diseases or manage their symptoms. Resveratrol can influence multiple inflammatory and non-inflammatory responses, protecting organs and tissues, thanks to its interaction with immune cells and its activity on SIRT1. This compound has anti-inflammatory, antioxidant, anti-apoptotic, neuroprotective, cardioprotective, anticancer, and antiviral properties, making it a potential adjunct to traditional pharmaceutical therapy in public health. This review aims to provide a comprehensive analysis of resveratrol in terms of active biological effects and mechanism of action in modifying the immune cellular response to promote human psychophysical health.

## 1. Introduction

Diet and proper lifestyle play a key role in maintaining well-being and preventing diseases. In recent years, due to their potential antioxidant activity, the use of polyphenols as adjuvants in mitigating risk factors for various disabling diseases has been evaluated [1]. Polyphenols, organic compounds abundant in plants, microalgae, fungi, and yeasts, represent an interesting and relatively new field of study for nutraceuticals and nutrition. Several studies show that the consumption of polyphenols can contribute to the maintenance of health by regulating metabolism, weight, chronic diseases, and cell proliferation [2,3]. Experimental studies, clinical trials, and epidemiological research highlighted that polyphenols exert antioxidant and anti-inflammatory activities that could have beneficial effects on cardiovascular diseases, neurodegenerative disorders, cancer, and obesity [4].

According to the World Health Organization (WHO), 52% of premature deaths in 2012 were attributable to non-communicable diseases (NCDs), with more than 75% related to cardiovascular diseases, cancer, diabetes, and chronic respiratory diseases, which represent one of the main public health concerns in the world. A modification in training, lifestyle, and diet (by eating more fruits and vegetables) may help to reduce the onset of these pathologies [5]. The European Food Safety Authority (EFSA) has stated that the consumption of 5 mg/kg/day of polyphenols could help in the prevention of such diseases [6].

Resveratrol (RSV) (3,4,5-trans-trihydroxy-stilbene), a phenolic compound found in red fruits and berries, in particular in grapes pulp, skin, seeds, and stems [7], exhibits a wide range of highly active biological effects to counteract the onset of many diseases or to manage their symptoms [8]. One of the possible mechanisms by which resveratrol plays its role in maintaining health is the suppression of inflammatory reactions by acting on immune cells [9]. RSV has antioxidant, anticarcinogenic, anti-inflammatory, neuroprotective, cardioprotective, and anti-aging properties [10,11]. This natural flavonoid can mitigate toxicities related to chemo-radiation therapy in normal tissues [12] and enhance migration of cells toward injured areas, which is important from therapeutic standpoints [13]. It also has modulatory effects on the tumor microenvironment [14]. Furthermore, this polyphenol is also involved in the maintenance of liver metabolism [15], interacts and strengthens the immune system [16], and acts on the nervous system [17]. Some studies report the neuroprotective effect of RSV against neurodegeneration, which sees its genesis in an impaired mitochondrial function [18]. RSV crosses the blood-brain barrier to counteract oxidative damage in hippocampal neurons and glial cells [19]. It has been described that RSV can activate the Silent Information Regulator 1 (SIRT1), a type of histone deacetylase, to protect neurons against apoptosis, inflammation, and oxidative stress in the treatment of neurological disorders such as Parkinson’s disease [20]. It also modulates cholinesterase activity and preserves dopaminergic neurons, which enhance learning and memory. It has been shown that RSV can reverse social dysfunctions. Recent studies have suggested the effect of RSV in improving panic and anxiety behaviors [21,22]. Recent studies have shown that stilbenes, in particular *trans*-RSV [23] and its glucoside, are able to bring numerous benefits to human health, showing antioxidant, cardioprotective, and antitumoral effects [24,25]. Several studies prove the use of this phenolic compound in the treatment and prevention of diseases with tumor etiology, helping to stop uncontrolled cell growth and consequently suppress cancerous neoformations [26]. The cardioprotective activity of RSV is associated with the inhibition of platelet aggregation and oxidation of low-density lipoproteins (LDL), followed by increased artery vascular relaxation [9]. Several clinical studies have shown that RSV may be useful for diabetic patients. The therapeutic effect of this compound on diabetes is complex and includes several beneficial functions [27]. The use of RSV, alone or in combination with modern antidiabetic therapies, may become an effective treatment for diabetes mellitus or its complications [9]. Since RSV is a natural antibacterial agent, researchers are increasingly using it to treat acute inflammation and chronic illnesses [28].

The biological properties of RSV tend to be closely related to a hormetic effect: low levels are associated with positive effects, while excessive amounts may be toxic [29]. Its hormetic qualities may be ascribed to its dose-dependent biphasic impact on cellular redox state, which is an antioxidant at low doses and a prooxidant at high concentrations [29,30]. Despite a considerable number of human and animal studies demonstrating the therapeutic and protective qualities of RSV [31,32,33,34], there are not enough clinical trials revealing the contentious and deleterious effects of this flavonoid [35].

The purpose of this review is to provide a detailed assessment of the active biological effects and of the mechanism of action of RSV in modulating the immune cellular response to improve human psychophysical health.

## 2. Resveratrol and Immune Cells

RSV can enter the cell via passive diffusion, mediated endocytosis, or via transporter proteins, binding to specific receptors, such as integrin receptor αvβ3 [36,37]. It contributes to modulating innate and adaptive immunity, stimulating the activation of macrophages, T cells, and natural killer (NK) cells, and cooperating in the inhibitory regulation of CD4+ CD25+ T cells [38]. This phenolic molecule can eliminate reactive oxygen species (ROS), inhibit the oxygen cycle (COX), and activate many anti-inflammatory pathways, including SIRT1. SIRT1 disrupts the Toll-like receptor (TLR)-4/nuclear factor-kappa B (NF-κB)/signal transducer and activator of transcription (STAT) signal, decreasing the production of cytokines by immune cells and of pro-inflammatory mediators derived from macrophages or mast cells, such as platelet activation factor (PAF), tumor necrosis factor (TNF)-α, and histamine [39]. SIRT1 is also involved in several molecular events, including metabolism, cancer, embryonic development, and immunotolerance [40,41]. Ablation of SIRT1 leads to increased activation of T cells and the onset of spontaneous autoimmune diseases. 

**Figure 1:**
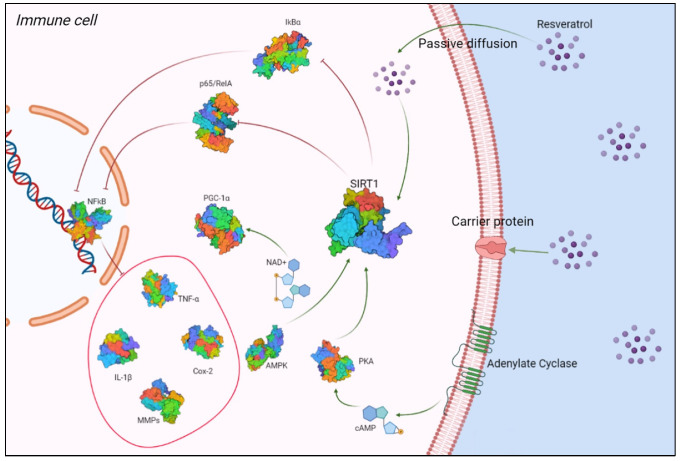
Interaction between RSV and SIRT1. Created with BioRender.com (Web online version 2021. Accessed on 28 December 2021).

Due to its ability to activate SIRT1, RSV is able to relieve inflammatory symptoms in several experimental models of autoimmune diseases, such as type I diabetes, encephalomyelitis, and rheumatoid arthritis [42]. Activation of SIRT1 by RSV results in inhibition of p65/RelA acetylation, an NF-κB member, which is the main regulator of leukocyte activation and the signaling of inflammatory cytokines. This mechanism reduces the NF-κB-induced expression of inflammation factors, such as TNF-α, interleukin (IL)-1, IL-6, metalloproteinases (MMP)-1 and -3, and COX-2 [42] (Figure 1).

RSV crosses the membrane by three mechanisms, namely passive diffusion, endocytosis via lipid rafts, or by carrier-mediated transport, binding to receptors such as integrin αvβ3. Once in the cell, it activates SIRT1, which inhibits RelA acetylation and promotes inhibitor protein-B (IkB) degradation, lowering NF-kB-induced production of TNF-α, IL-1, IL-6, MMPs, and COX-2. Protein kinase A (PKA) is activated by cyclic adenosine monophosphate (cAMP) levels, which activate SIRT1. SIRT1 activity is controlled by AMP-activated protein kinase (AMPK), which regulates cellular levels of nicotinamide adenine dinucleotide (NAD+). Increased NAD+ levels activate SIRT1, which promotes deacetylation and activation of peroxisome proliferator-activated receptor-gamma coactivator 1-alpha (PGC-1)α, which is downstream of AMPK.

The development of tumors and inflammation are linked [43]. As a result, anti-inflammatory drugs can help to prevent cancer from growing. RSV has been demonstrated to have cytotoxic and anti-tumor effects in vitro against a variety of cancers, including breast, ovarian, stomach, liver, thyroid, and prostate cancers. RSV can act on the signal transduction pathways involved in angiogenesis, metastasis, apoptosis, inflammation, and cell proliferation [44], affecting cancer development and progression. RSV was used to counteract colorectal cancer in a mouse model in a 2020 study. Treatment increased in anti-inflammatory cells CD4+ T regulatory (Tregs) and CD4+ IL-10+, induced a decrease in pro-inflammatory cells T helper (Th)1 and Th17, leading to a deceleration of colorectal cancer growth [43]. The study of immunomodulation of the tumor microenvironment is becoming increasingly essential in the treatment of cancer patients. Several non-tumor cells, mostly endothelial cells, carcinogenic fibroblasts, and immune cells, primarily related tumor macrophages, cytotoxic T cells, NK cells, B cells, Treg cells, and dendritic cells (DCs), make up the tumor microenvironment [45]. These immune cells play a key role in the pathophysiology of cancer growth and progression. RSV has recently been postulated as an immunomodulatory drug capable of stimulating immune cells in the tumor microenvironment or sensitizing tumor cells to immune cell cytotoxicity [42]. RSV has been proven to increase the production of anti-tumor cytokines, including interferon (IFN)-γ and TNF-α, as well as to decrease the production of tumor growth factor (TGF)-β. It can also encourage CD4+ T cells and macrophages to polarize towards anti-cancer cells while reducing the invasion and polarization of immunosuppressive cells [46].

### 2.1. Macrophages

Macrophages, immune cells that differentiate from monocytes, cooperate in the balance of innate and adaptive immunity. These cells form a heterogeneous pool of cells with a broad spectrum of biological activities depending on where they reside and the external inputs they receive. These immune cells have a wide range of pattern recognition receptors (PRRs), allowing them to identify specifically conserved molecular patterns associated with pathogens (PAMPs), which are exclusively present on agents such as viruses, bacteria, parasites, and fungi [42]. The main members of the PRR families are TLRs transmembrane proteins, C-type lectin receptors (CLRs), cytoplasmic nucleotide oligomerization domain (NOD)-like receptors (NLRs), and gene I-like receptors (RIG-I) of the RNA helicase induction (RLR). Macrophages produce anti-inflammatory cytokines, such as IL-10 and TGF-β, and inhibit inflammatory pathways mediated by TLRs [47]. TLRs initiate the signal cascade in innate and adaptive immune pathways. These highly phylogenetically conserved receptors [48,49] from some invertebrates, such as ascidians [50] to mammals, passing through all classes of vertebrates [51,52], represent a family of trans membrane proteins with two binding sites: an extracellular domain engaged in the recognition of potentially harmful antigens, both exogenous and endogenous, and an ectodomain that activates responses, determined by antigen binding. 

RSV regulates the expression of TLR-4. Therefore, RSV can interact with TLR-mediated inflammatory responses, and chronic diseases related to TLR activation, including obesity, type 2 diabetes mellitus (T2DM), fatty liver disease, Crohn’s disease, rheumatoid arthritis, cardiovascular disorders, and neurodegenerative disorders [53]. It inhibits TANK-binding kinase1 (TBK1), a protein kinase serine/threonine involved in the antiviral response of innate immunity. Further studies have shown that RSV exerts an anti-inflammatory action by interacting with TLR-4, Tumor necrosis factor receptor-associated factor 6 (TRAF6), mitogen-activated protein kinase (MAPK), and Protein kinase B (PkB) pathways in lipopolysaccharides (LPS)-induced macrophages [54]. SIRT1 has a direct regulatory role in the functions of macrophages during inflammation both in the secretion of cytokines and in the expression of cell adhesion molecules as intracellular cell adhesion molecules 1 (ICAM-1) [55]. RSV also plays a key role in protecting the endothelium from inflammation, reducing the production of large amounts of colony-stimulating factor (GM-CSF), a pro-inflammatory cytokine essential for the differentiation and activation of pro-inflammatory macrophages and which represents a specific marker of the formation of atheromatous plaques [56]. Therefore, RSV can modify cell morphology, gene expression, ligand–receptor interactions, signaling pathways, and foam cell formation, involved in atherosclerosis [57]. In addition, RSV modulates the immune system response by affecting cellular levels of prostaglandin E2 (PGE2) which plays an important role in regulating the immune response [58]. Finally, RSV up-regulates the secretion of chemokines, such as COX-2, in various inflammatory diseases [59]. By activating and stimulating macrophages and the production of IL-1 and IL-6, RSV also facilitates the systemic response to lesions and cooperates with tissue regeneration [42].

### 2.2. NK Cells

NK cells represent about 15% of all circulating lymphocytes and play a critical role in the defense against pathogens and cancer [60]. NK cells express various PRRs such as TLRs, NLRs, and RLRs. Activated NK cells release IFN-γ, GM-CSF, TNF-α, or cytotoxic granules directed towards a target cell. NKs kill target cells through different mechanisms: inducing cell lysis, releasing cytoplasmic granules containing perforin (Prf1) [61], or by the secretion of several effector cytokines, such as IFN-γ, IL-5, IL-10, IL-13, and GM-CSF, which trigger apoptosis in the target cell. NK cells also secrete a variety of chemokines including the C-C chemokine ligand (CCL), such as CCL2, CCL3, CCL4, CCL5, monocyte chemoattractive protein (MCP-1), macrophages inflammatory protein (MIP-1), chemokine X-C chemokine ligand (CXCL-1), and IL-8. NKs, by interacting with other immune cells such as dendritic cells in areas of inflammation, modulate the innate and adaptive immune response and promote the T-cell response against tumors [62]. 

RSV exerts simultaneous effects on NK and T cells [63]. It helps to increase the cytotoxicity of NK cells in vitro and in vivo, suggesting that RSV could be used as an adjuvant for cancer immunotherapy [63]. An increase in the tumor lysis activity of NK cells was observed in a study evaluating the anti-infective properties of RSV in a mouse model of acute pneumonia [64,65]. The RSV group showed increased alveolar macrophage infiltration, increased NK cell activity infiltration, reduced lung bacterial load, and decreased mortality. Spleen NK cells isolated from rats pre-treated with RSV were more effective in inducing cell death against YAC-1 cultured target tumor cells [42].

### 2.3. Dendritic Cells

DCs are a heterogeneous family of antigen-presenting immune cells (APC) [66]. These cells are primary sentinels and share many characteristics with macrophages, which may also present antigen on major histocompatibility complex (MHC) class II molecules, although not as effectively as DCs. RSV exerts potent immunomodulatory effects on DCs function [67]. 

RSV inhibits the expression of the co-stimulating molecules CD80 and CD86 and MHC class II molecules, leading to the suppression of phenotypic DC maturation, reducing the DC capacity to stimulate the proliferation of naive allogeneic CD4+ T cells. Interestingly, due to the many molecular targets of RSV, many of which are involved in optimal DC maturation, RSV appears to exert even more potent immunosuppressive effects during the DC differentiation process. DCs from human peripheral monocytes incubated at various concentrations of RSV led to the creation of “alternatively” differentiated DCs, unable to respond optimally to maturation stimuli, even in the absence of RSV [68]. In addition, to inhibit surface expression of the co-stimulating molecules, these DCs showed increased surface levels of transcripts similar to immunoglobulin-like transcript (ILT)-3 and ILT-4, two molecules closely associated with the tolerogenic DC function [69]. Moreover, these DCs can secrete greater amounts of IL-10 [68]. 

### 2.4. T and B Lymphocytes

T and B lymphocytes cooperate with the APC in the immune response. However, once adaptive immune responses are triggered, Th1 and Th17 migrate from lymphoid tissue to the circulation, infiltrating infected sites, and producing their cytokines, strengthening the activity of macrophages and neutrophils. Both innate and adaptive immunity can control inflammation and develop self- and nonself-discrimination. By T-cell receptor (TCR), T-cells are also involved in cellular recognition processes, along with proteins of the MHC, inducing possible apoptosis in a negative selection [42]. To maintain immune balance, Tregs play a role in preventing the onset of autoimmune diseases and modulating the inflammatory response. Uncontrolled and abnormal activation of T cells is implicated in many autoimmune diseases, such as insulin-dependent diabetes, rheumatoid arthritis, systemic lupus erythematosus, and multiple sclerosis [70]. 

RSV may inhibit the activation of T cells and reduce the production of cytokines, preventing the autoimmune progression of the disease. Experimental studies have shown that mice with arthritis treated with RSV showed a significant reduction in the disease. Histomorphological evaluations have shown that the immune cells infiltrated into the joints were significantly reduced in mice treated with RSV compared to control mice, concluding that RSV may prevent the development of arthritis [71]. Th17 cells are the main initiators of pro-inflammatory responses, recruiting neutrophils and macrophages at the site of infection, and through the production of IL-17 play an important role in defense against extracellular pathogens. Th17 also secretes IL-23, which controls the survival and maintenance of the Th17 phenotype and is responsible for the crosstalk between innate and adaptive immunity [72]. Th17 cells produce IL-22 which, similarly to IL-17, is involved in many infectious and inflammatory disorders. In a clinical trial, male T2DM patients with hypertension were randomized to receive 350 mg/day for the first six months and 700 mg/day for the next 6 months of conventional grape extract without RSV, grape extract with RSV, or a placebo of maltodextrin [73]. At the end of the study, IL-6 was significantly decreased only in the group administered grape extract with RSV. In the placebo group, adiponectin and IL-10 decreased significantly, while the IL-6/IL-10 ratio increased. Levels of several pro-inflammatory mediators (TNF-α, IL-8, and IL-1β) were significantly decreased in the group taking grape extract with RSV compared to placebo [74]. In addition, the administration of RSV suppresses the CD4+ CD25+ cell population among cells CD4+, reduces TGF-β secretion, and enhances IFN-γ expression in CD8+ T cells both ex vivo and in vivo, resulting in immunostimulation. RSV can inhibit T cells activation in autoimmune diseases, and reduce Tregs suppressive function to inhibit tumor growth. 

**Figure 2:**
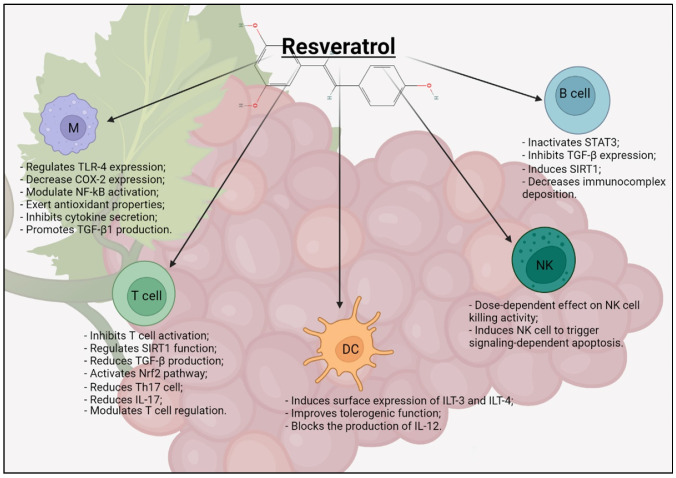
Scheme of RSV effect on immune cells. M = macrophage; DC = dendritic cell; NK = natural killer cell. Created with BioRender.com (Web online version 2021. Accessed on 28 December 2021).

RSV induces the mitogenic proliferation of B cells at lower concentrations but inhibits it at concentrations higher than 10μM. RSV does not appear to affect IgG or IgM production, but does induce a dose-dependent increase in the Bcl-2 expression, proteins that govern the permeability of the outer mitochondrial membrane, and can be either pro-apoptotic or anti-apoptotic [75]. All effects of RSV on immune cells are summarized in Figure 2.

## 3. Resveratrol and COVID-19

In recent years, several viral pathologies have followed one another, such as Severe Acute Respiratory Syndrome (SARS-CoV) in 2003, H1N1 (swine fever) in 2009, and Middle East Respiratory Syndrome (MERS-CoV) in 2012. Since November 2019, a new type of Coronavirus has caused a severe form of pneumonia, spreading rapidly worldwide since it was first detected in China, so much so that on 11 February 2020, the WHO declared the pandemic state. The disease induced by SARS-CoV-2 has been called Coronavirus Disease 19 (COVID-19) [75,76]. This infection causes several symptoms: from a dry cough to fever, from dyspnea to bilateral pneumonia. Patients with severe symptomatology can suffer acute respiratory distress syndrome, leading to admission to intensive care and, in several cases, even death [77]. Many studies indicate that natural compounds have biological activities that counteract the onset of many viral infections, enforcing the immune response, limiting inflammation, counteracting the action of free radicals, and playing a potential viricidal role [78]. 

Scientific evidence shows that RSV can exert viricidal action against several viruses. Several inflammatory mediators, such as NF-kB, TNF-α, and MMP-9, were observed to be higher in chronic obstructive pulmonary disease (COPD) patients than in healthy controls and were reduced after treatment with RSV [79,80,81]. The severity of COVID-19 is a consequence of lung inflammation and injury caused by the viral infection but also of an excessive inflammatory reaction, related to the inefficiency of immune response triggered to control the virus: the cytokine storm [82]. RSV can interfere with viral replication through the inhibition of viral gene expression, viral nucleic acid, protein synthesis, and downregulation of various cellular transcription and signaling pathways [65]. RSV can also reduce the cytokine storm, acting on immune cells (such as T cells, DCs, and macrophages) and limiting the lung-associated parenchyma injury. These properties have been demonstrated in infections induced by the influenza virus, respiratory syncytial virus, human rhinovirus, and MERS-CoV [83]. Vero cell studies have shown that RSV significantly inhibits the dose-dependent replication of SARS-CoV-2 with an effective concentration (EC)50 of 4.48 mM [84]. Adding 50 mM of RSV to cells after the virus absorption phase inhibited SARS-CoV-2 replication by 98%, suggesting that the molecule may also exert an effect on viral entry into cells [85]. In Vero E6 cells, plaque-assessed viral replication was reduced by 50% and 90% respectively at RSV concentrations of 66 and 119 mM. Significant antiviral activity was observed up to 40 h post-infection, a time-point corresponding to approximately five cycles of viral replication, demonstrating the long-lasting antiviral effect of the compound. 

RSV was then evaluated for antiviral activity in human primary bronchial epithelial cells obtained from healthy individuals [86]. In addition to exhibiting direct antiviral activity, RSV showed inhibitory functions on the pathogenetic mechanism involved in the severity of COVID-19 [87]. These include the activation of unregulated NLRP-3 inflammasome, dysfunction of the renin-angiotensin system, and stimulation of the quinine-callicrein system. These inhibitory functions are mediated by the induction of the SIRT1 protein that overregulates the expression of the angiotensin-converting enzyme (ACE)2 protein [83]. NLRP-3 inflammasome is a multiprotein complex that exists as a latent monomer in quiescent cells. Physiological activation of NLRP-3 by stressors or pathogenic microorganisms facilitates the release of pro-inflammatory cytokines and promotes the induction of an adaptive immune response against bacteria and viruses. However, its uncontrolled dysregulated activation is involved in the pathogenesis of several inflammatory disorders [88]. SARS-CoV-2 can activate NLRP-3 directly through a viral protein viroporin 3a, and indirectly through overproduction of IL-1β, a known causal factor for the more serious complications of COVID-19 [89]. 

Autophagia can control activation of NLRP-3 inflammasome through interactions with a variety of immune signaling pathways and the removal of endogenous inflammatory agonists. RSV can activate SIRT1, a deacetylase that exerts protective effects on a variety of cellular functions, including apoptosis [90]. In an experimental lung study of mice infected with a respiratory syncytial virus, SIRT1 promoted autophagy-mediated processes leading to the activation of dendritic cells, the presentation of viral antigen to T cells, and an effective antiviral immune response [10]. Thus, through downregulation of the excessive inflammatory response demonstrated in other viral infections [91] and upregulation of SIRT1 activity, RSV can inhibit NLRP-3 activation, leading to autophagy [92].

A study by ter Ellen et al. (2021) [86] confirmed that RSV has a potent antiviral effect against COVID-19. This phenolic compound greatly reduced the replication of SARS-CoV-2 not only in Vero E6 cells, but also in a human bronchial epithelial cell model. Moreover, it actively interferes with the infectious replication cycle of the virus and exerts antiviral activity up to about five replication cycles of SARS-CoV-2 in vitro. The study hypothesizes that RSV interferes with the early stages of virus replication, thereby reducing the chance of productively infecting a cell. 

**Figure 3:**
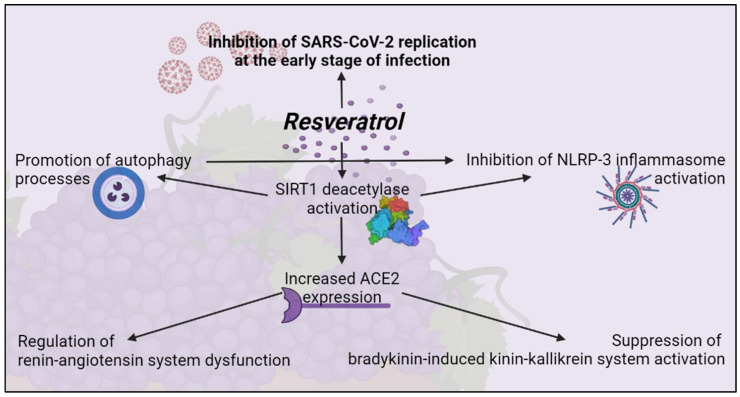
RSV inhibits SARS-CoV-2 replication at an early stage of infection. Created with BioRender.com (Web online version 2021. Accessed on 28 December 2021).

In addition to the antiviral properties targeting SARS-CoV-2 replication, RSV may also be suitable for moderating the exacerbated inflammatory response seen in patients with COVID-19 [93]. RSV, as an antioxidant, is found to prevent the formation of reactive oxygen species (ROS), prevent epithelial airway remodelling by upregulation of SIRT1, and superoxide activate dismutase (SOD) [94]. Due to the low bioavailability of RSV after oral administration, an effective antiviral dose against respiratory viruses in humans is unlikely to be achieved by this route of administration. Therefore, alternative modes and routes of administration have been investigated. Promising strategies for antiviral treatment are the use of RSV aerosol suspension sprays, dried microparticles co-spray, or nanotechnological approaches [95]. While improving bioavailability, some of these techniques allow direct and local pharmacological administration of RSV to the primary active site of replication of SARS-CoV-2 by inhalation-based systems, currently considered a favorable future strategy for the treatment of COVID-19 [96]. Therefore, RSV might not only be a promising candidate for inhibiting viral replication at the onset of infection, it may also alleviate the symptoms of the disease later in infection (Figure 3).

## 4. Resveratrol and Metabolic Syndromes

Obesity represents an authentic epidemic: the prevalence of overweight (body mass index BMI ≥ 25 kg/m^2^) and obese (BMI ≥ 30 kg/m^2^) people has increased since the 1970s [97] and in 2016 about 39% of the world’s adult population was overweight [98]. Obesity has serious health effects, increasing the risk of developing type 2 diabetes, cardiovascular disease, dyslipidemia, Alzheimer’s disease, and even certain cancers [99]. Reducing obesity requires major lifestyle and dietary changes to avoid surgery or medication.

RSV showed several positive effects on the management of obesity, inducing mitochondrial biogenesis and oxidative phosphorylation via the activation of peroxisome proliferator-activated receptor γ coactivator-α (PGC-1α) via SIRT1 deacetylase, leading to improved insulin sensitivity [100], and by increasing the expression of SIRT1, a reduction in the size of adipocytes, showing an anti-lipolytic effect [101]. A recent study on a mouse model fed with a high-fat diet showed that RSV supplementation can improve the composition of the intestinal microbiota and enrich the pathways involved in generations of small metabolites, despite the low bioavailability of this polyphenol, contributing significantly to the prevention of metabolic syndrome [102]. 

**Figure 4:**
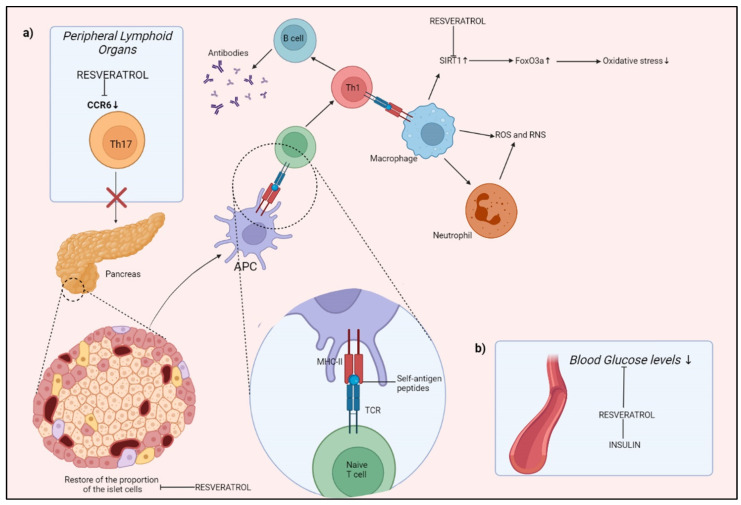
RSV action on DM. (**a**) action on CCR6, (**b**) action on insulin. Created with BioRender.com (Web online version 2021. Accessed on 28 December 2021).

Diabetes mellitus (DM), a group of chronic metabolic diseases characterized by hyperglycemia, is one of the most serious consequences of obesity, caused mainly by a total or partial dysfunction of the Langherans islands of the pancreas, responsible for the production of insulin, the hormone that regulates blood glucose levels. In 2017, the disease affected about 451 million people, and this number could increase to 693 million by 2045 [103]. Often, the various vascular complications caused by hyperglycemia, together with oxidative stress and inflammation, are the primary reason for morbidity and mortality associated with diabetes [104]. RSV, however, has potent antioxidant and anti-inflammatory activities, acting directly on lipid peroxidation and removing superoxide free radicals. By activating SIRT1, RSV induces the deacetylation of the transcription factor Forkhead box O (FoxO), involved in cell apoptosis, and increases the expression of SOD, which has a key antioxidant role [105]. The absorption of β cell antigens by islet resident DCs, presentation to naive T cells, and promotion of Th1 development will activate B lymphocytes, which will create autoantibodies against beta cells. Th1 will also trigger macrophage and neutrophil migration to the islet, which will increase ROS and enhance β cell death. RSV works through: (a) binding to C-C chemokine receptor (CCR)6, which inhibits Th1 cell migration; (b) creating a complex with insulin, which stimulates glucose intake. RSV also reduces ROS and boosts antioxidant capacity by inhibiting apoptotic cell damage during oxidative stress via SIRT1. RSV also aids in the regeneration of beta cells in the pancreas (Figure 4).

A clinical study on thirteen patients with type 1 diabetes was performed by Movahed et al. in 2019. These subjects were given RSV in 500 mg capsules twice daily for 60 days. Fasting blood glucose and hemoglobin A1c levels were significantly reduced with the supplementation of RSV, also leading to a decrease in malondialdehyde, a marker for oxidative stress. This confirms the strong antidiabetic and antioxidant effects of RSV, which can be considered a valid aid for the prevention and management of metabolic syndromes [106]. Moreover, a meta-analysis of 11 individual studies showed that RSV consumption significantly reduced fasting glucose, insulin, glycated hemoglobin, and insulin resistance levels in the subgroup of diabetes patients [107]. However, further clinical and preclinical evidence with larger samples, together with further studies to improve its bioavailability, are needed to confirm the efficacy and safety of this polyphenol in such delicate pathologies.

## 5. Resveratrol and Neuroinflammation

Neuroinflammation is the brain response to an insult, infection, or disease, which aims to remove or inactivate potentially damaging agents to brain tissue. This response is mediated by the interaction between two cellular systems: the central nervous system (CNS), whose activation leads to the triggering of certain immune cells of the hematopoietic system [108]. Neuroinflammation is one of the major contributing factors in exacerbating neurodegeneration and depression, which, especially in combination with chronic stress, can increase the level of pro-inflammatory cytokines, such as TNF-α and IL-1β, leading to the development of diseases such as Alzheimer’s disease (AD) and Parkinson’s disease (PD) [109,110].

Many studies have shown the neuroprotective and immunomodulatory role that RSV plays against neurodegenerative diseases [111,112]. RSV is one of the major activators of SIRT1, a nuclear protein that deacetylates transcription factors that govern metabolic central pathways, whose key role in the management of AD has been widely evaluated. When it is overexpressed in the brain, it reduces this neurodegenerative disease, as it directs the division of amyloid precursor protein (APP) from β-amyloid production through the activation of α-secretase, and deacetylates the tau protein, thereby reducing the formation of neurofibrillary tangles [113,114,115]. 

In a 2016 study in which a memory and learning deficit was induced in a mouse model by microinfusion into the hippocampus of β-amyloid_1-42_, the main constituent of amyloid plaques that forms during the exerts of AD [116], higher dose (40 mg/kg) of RSV was shown to significantly improve the memory of rats, and to decrease the expression of apoptotic and inflammatory cytokines, such as IL-1β and IL-6, acting on T cells. The inhibitory effect of RSV counteracts the activity of phosphodiesterase-4 (PDE-4), which controls intracellular levels of cAMP [117].

In a rat model with a spared nerve injury, performed to simulate symptoms of neuropathic pain, RSV has shown neuroprotective effects, inhibiting the inflammatory response of neuropathic pain, as well as relieving mechanical allodynia, one of the main symptoms of this chronic disease, through an administration of 300 μg/day, improving autophagy and downregulating the expression of triggering receptors expressed on myeloid cells 2 (TREM2) in microglial cells, identified as one of the critical factors in inflammation in the nervous system [118]. 

A clinical study in patients with AD showed that after 52 weeks of RSV administration (up to 1 g twice daily), the levels of MMP-9, a matrixin involved in the degradation of the extracellular matrix, in the cerebrospinal fluid of these patients were significantly decreased. MMP-9 appears to be implicated in several brain pathologies, such as neurodegeneration and neuroinflammation [119] because it regulates the permeability of the blood–brain barrier through the release of cytokines and free radicals. The reduction in this enzyme shows that RSV administration reduces brain permeability and, as a result, the infiltration of leukocytes and other inflammatory agents into the brain, as well as inducing an adaptive immune response that may increase brain resilience to amyloid formation [18].

## 6. Resveratrol and Infertility

Infertility is a widespread condition in industrialized countries where it affects 15–20% of couples [120]. It is defined as the inability to reach a spontaneous pregnancy after at least one year of regular and unprotected sexual intercourse [121]. Male infertility may include abnormal sperm parameters (oligozoospermia, asthenozoospermia, teratozoospermia) or a combination of all three (oligo-astheno-teratozoospermia), or azoospermia [122]. Male infertility causes can be divided into four categories (endocrine and systemic illnesses, primary testicular problems in spermatogenesis, and sperm transport disorders), with idiopathic infertility accounting for up to 25% of cases [122]. Most studies on infertility treatment, both in vitro and in vivo, have focused on: mechanisms of oxidative stress that could cause: (1) lipid peroxidation, with altered membrane fluidity and permeability resulting in decreased sperm motility and reduced sperm ability to interact with the egg cell; (2) protein modification resulting in reduced adenosine triphosphate (ATP) production; and (3) increased fragmentation of sperm DNA [123].

Male infertility may be associated with testicular immune microenvironment disorders. The testicular immune microenvironment consists of common immune cells associated with other cells of the organ, all involved in testicular immunity. These cells include macrophages, T cells, DCs, and mast cells associated with Leydig cells and Sertoli cells (SCs). Several studies have shown an overproduction of pro-inflammatory cytokines dependent on alterations of this microenvironment as in the case of varicocele [124]. The anti-inflammatory action of RSV could inhibit this cytokine hypersecretion, counteracting infertility.

A clinical study evaluated the protective effect of RSV in counteracting infertility damage induced by benzo(a)pyrene (BaP), a highly toxic and carcinogenic polycyclic aromatic hydrocarbon originating from cigarette smoking, smoke from some factories, and even from burned food [125]. The study was conducted on 30 healthy men with an average age of around 32 years at the Division of Andrology and Endocrinology of the University of Catania. Sperm of 15 men were incubated with increasing concentrations of BaP (concentrations of 0, 15 and 45 μM/mL of BaP), at 37 °C. The effects of BaP on sperm motility and bio-functional parameters were assessed after the incubation period. Once BaP response concentration was established, the sperm of the remaining 15 men were exposed to only 15 μM/mL BaP (the lowest effective concentration) and/or 15 μM/mL RSV [126]. BaP resulted in a progressive decrease in sperm motility and abnormal compact chromatin. RSV showed its antioxidant and anti-inflammatory effect by decreasing the percentage of sperm with altered chromatin compactness. It also improved the total and progressive motility of sperm and lowered the oxidative stress indices [126].

Some studies [127] have shown that RSV appears to be able to enhance the estrogenic effects of hormones, and therefore is a modulator of female reproductive function. Estrogens are also secreted by human Leydig cells in the testicles where they play a paracrine regulatory function [128]. RSV is a phytoestrogen in females, with a chemical structure comparable to that of estrogens. RSV shows a cytoprotective effect on oocytes with antioxidant and antiapoptotic effects. In males, RSV affects reproductive function in three ways: (1) by improving testosterone production, (2) by triggering penile erection, and (3) by improving spermatogenesis [129]. This suggests a possible role of RSV in male fertility. This polyphenol activates antioxidant enzymes, such as catalase and SOD, involved in the lipid damage of human sperm during the cryopreservation process [130]. In addition, RSV shows an anti-inflammatory effect by decreasing the activity of inflammatory molecules, such as COX-2, inducible Nitric Oxide Synthases (iNOS), a linking enzyme between immune and endocrine cells [131,132,133,134,135], and B cell NF-kB [136]. Regulating anti- and pro-apoptotic mediators also protects cells from DNA damage and apoptosis [128]. Spermatogenesis, therefore, can be extremely sensitive to compounds that interfere with mitochondrial energy metabolism and respiratory control. Any alteration in the regulation of metabolic behavior of these cells may impair the normal development of spermatogenesis and, consequently, male fertility [137]. It has been proposed that mitochondria also play a role in this sperm degenerative process, thus ensuring that good quality meiotic products enter the spermatogenesis process to produce quality mature sperm [138]. Interestingly, RSV has been shown to increase the mitochondrial number (mitogenesis) and activity (ATP concentration) in several cell types, such as muscle cells [139] and granular cells [140]. RSV enhances mitochondrial function by activating SIRT1 [139]. Thus, it can regulate cell biology, metabolism, and fate at various levels [141,142,143].

## 7. Conclusions

**Figure 5:**
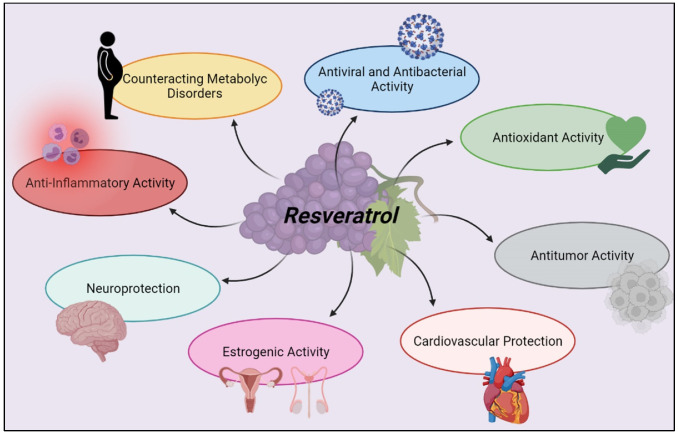
Summarizing scheme of RSV biological activities. Created with BioRender.com (Web online version 2021. Accessed on 28 December 2021).

The broad spectrum of action of RSV makes this phenolic compound able to counteract the onset of different diseases or to contain their symptoms. Thanks to the interaction that RSV has with immune cells and its action on SIRT1, this polyphenol can modulate different inflammatory and non-inflammatory responses, inducing protective effects on organs and tissues. The anti-inflammatory, antioxidant, anti-apoptotic, neuroprotective, cardioprotective, antitumor, and antiviral power make this compound a possible adjuvant of classic pharmacological therapies in public health management (Figure 5). New experimental and clinical studies are needed to improve the bioavailability of this nutraceutical and to evaluate the most appropriate route of administration to maximize its efficacy. In addition, broadening the sample size in the various clinical trials could provide a clearer and more complete picture of the efficacy and safety of RSV.

## Data Availability

Not applicable.

## Sample Availability

Samples of the compounds are not available from the authors.
